# An initial typology of approaches used by policy and practice agencies to achieve sustained implementation of interventions to improve health

**DOI:** 10.1186/s43058-024-00555-2

**Published:** 2024-03-05

**Authors:** Luke Wolfenden, Adam Shoesmith, Alix Hall, Adrian Bauman, Nicole Nathan

**Affiliations:** 1 School of Medicine and Public Health, College of Health, Medicine and Wellbeing, University of Newcastle, University of Drive, Callaghan, NSW 2308 Australia; 2 National Centre of Implementation Science (NCOIS), The University of Newcastle, Wallsend, NSW Australia; 3 Hunter New England Population Health, Hunter New England Local Health District, Wallsend, NSW Australia; 4 Hunter Medical Research Institute (HMRI), New Lambton Heights, NSW Australia; 5 School of Public Health, University of Sydney, Sydney, NSW Australia

**Keywords:** Sustainability, Typology, Evidence-based intervention

## Abstract

**Background:**

Scientific investigation of how to sustain the implementation of evidence-based interventions (EBI) is emerging. Sustaining the implementation of EBIs helps ensure their effects on improving health endure. External policy or practice agencies, such as government health departments, are often tasked with supporting individual organisations with sustaining their delivery of EBIs, for example, through financing, training or the provision of other supports. However, to our knowledge, the approaches taken by policy and practice agencies to support the sustainment of EBIs have not been consolidated, categorised and described as a typology.

**Main body:**

To improve conceptual clarity and support both research and practice, we developed an initial working typology of the practical approaches to sustain implementation of EBIs (i.e. sustainment) in order to improve long term health from the perspective of these agencies. The working typology includes three broad approaches. The first, termed ‘*Self-Sustainment*’, is when implementation of the EBI by an organisation (e.g. hospital, clinic, school) is expected to continue (sustain) in the absence of external (agency) support. The second, termed ‘*Static Sustainment Support*’, involves the provision of pre-defined external (agency) support to assist organisations to continue implementation of an EBI. The final approach is termed ‘*Dynamic Sustainment Support*’, whereby support provided by an external agency is dynamic (continues to be adapted) overtime to assist organisations continue implementation of an intervention which may itself also evolve.

**Conclusions:**

We describe the contexts and circumstances where each may be most appropriate in achieving sustained implementation and discuss their research and practice implications.

**Supplementary Information:**

The online version contains supplementary material available at 10.1186/s43058-024-00555-2.

Contributions to the literature

- We propose a working typology we hope will contribute to a shared language and understanding of approaches to support sustainment of EBIs to improve health impact.
- We propose definitions for three broad approaches to achieving sustained implementation developed from the perspective of agencies responsible for supporting the implementation and sustainment of health interventions.
- We describe the contexts suggested as most appropriate for each approach and discuss research and practice implications.
- This working typology will contribute to conceptual advancement for the field and, in doing so, help researchers advance the science of sustainment and policy makers and practitioners to better design and develop appropriate strategies to support successful sustainment.

## Background

Globally, there has been significant investment in the development of evidence-based interventions (EBI) to prevent or reduce the incidence of chronic disease [1]. The advent of implementation science has enhanced delivery of EBIs in clinical and community settings [2]. While sustaining individual health behaviour changes has been the subject of behavioural science research for decades, more recently, attention has shifted to EBI sustainment, that is, the ongoing use or implementation (and so benefits) of EBIs [3–5]. This shift acknowledges the challenge of sustaining the routine delivery of EBIs following their initial implementation. For example, a comprehensive review of 125 empirical studies of public health and clinical interventions found that only 23% of implemented programs were sustained at least 2 years following initial implementation [3]. Similarly, a 2020 review which examined the sustainment of school-based health promotion interventions found that of the 18 included interventions, none were sustained in their entirety (i.e. all components) following the withdrawal of external implementation support [6].

A range of factors impede the sustainment of EBIs [4, 6, 7]. Undertaking research to better understand and address these may help to guide future sustainment efforts. As is often the case in emerging scientific disciplines, undertaking research to advance understanding and conceptual clarity is impeded through inconsistent use of key terminology [2, 8, 9]. Reviews of the literature suggest this is the case for the ‘science of sustainment’, with a diversity in definitions of sustainability in the literature [10]. Indeed a criticism of previous sustainment research is the poor and varied articulation of this concept [4, 9, 10]. As such, improving the clarity of sustainability terms and concepts is a priority for this field of research [10]. A well accepted, current, utilised [4, 11–15] and comprehensive definition of sustainability is ‘After a defined period of time, the program, clinical intervention and/or implementation strategies continue to be delivered and/or individual behaviour change is maintained; the program and individual behaviour change may evolve or adapt while continuing to produce benefits for individuals/systems’ [10].

This definition captures both the maintenance of changes to health or health behaviour of individuals receiving an EBI and of the sustainment of the implementation of an EBI. It also acknowledges that sustainability is often dynamic and may require adaptation to the EBI or support strategies to ensure its continued implementation. While the definition by Moore et al. [10] draws together key concepts of sustainment, limited work has been undertaken to describe approaches to achieve it. Such work would be particularly useful for external policy or practice agencies, such as governmental departments or coalitions, responsible for supporting organisations implement and sustain EBIs. For example, the New South Wales (Australian) local Health Promotion Units support the implementation and sustainment of government prioritized health promotion interventions in clinical (e.g. hospitals) and community (e.g. schools) organisations. What this support entails is usually determined by a range of factors including the availability of resources, characteristics of the EBI or the organisations delivering them. Characterising the approaches to supporting sustainment and reflecting on the circumstances where they may be most appropriate may be particularly useful given the current lack of empirical guidance for policy agencies. It may also guide future research to better understand and test the efficacy of different policy approaches.

In this manuscript, we aim to expand on the conceptual thinking relating to sustainability and propose an initial typology focussed on approaches to sustain the implementation of EBIs. It is undertaken from the perspective of agencies responsible for providing external monetary or non-monetary forms of support to facilitate implementation and sustainment of EBIs in clinical and community organisations. It also specifically draws on the perspectives and experiences of the authors who lead agencies responsible for supporting the implementation and sustainment of jurisdiction-wide health promotion intervention in Australia. It is hoped the typology will help contribute to a shared language and understanding of the pathways in which sustainment support may best be provided. In doing so, the paper aims to stimulate further work of researchers, policy makers and practitioners to better understand and support successful sustainment of EBIs.

## Proposed working typology

Typologies are tools to aid conceptual understanding and have been used to classify heterogeneous phenomena across a range of scientific fields, including behavioural and implementation science [16, 17]. To improve conceptual clarity, avoid homonymy and support both sustainability research and practice, we propose the following working typology of approaches taken by external agencies to support organisations to sustain EBIs to improve health (Additional file 1). The working typology is proposed following the consideration of the (i) Moore definition [10]; (ii) current models of sustainability [4, 18–20]; (iii) reviews of sustainability research [3, 6, 9], particularly those by Shelton and colleagues [4] and Stirman and colleagues [3]; and (iv) discussion and debate among the author team and our colleagues. The typology acknowledges perspectives that sustainment can occur in the absence of external support and so is influenced by factors such as how well delivery of an EBI has become routine, or institutionalised during the initial implementation period, or with external monetary or non-monetary support provided after initial implementation. For example, this may include but is not limited to the use of on-going prompts or reminders, the provision of resources to upskill and sustain intervention delivery, obtaining executive or leadership endorsement and commitment or implementing systems to continually monitor, evaluate and provide feedback on intervention delivery [4]. It incorporates perspectives articulated in models such as the ‘*Exploration, Preparation, Implementation, Sustainment (EPIS) Framework*’ [21] where sustainment was viewed as the end of an implementation process, where the EBI and strategies to support it are largely fixed and fidelity of delivery is prioritised. It also accommodates more contemporary perspectives articulated by the ‘*Dynamic Sustainability Framework*’ [5] and other reviews [4] where EBIs are dynamic, and sustainment is supported in the contexts of its evolution and improvement.

**Fig. 1:**
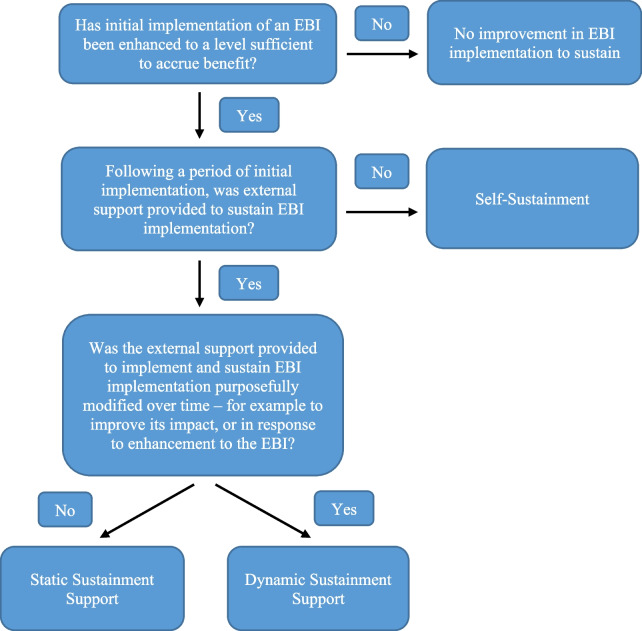
Decision tree for classifying approaches to achieve EBI sustainment

Specifically, the typology uses two key classification criteria born out of the existing definitions, models and reviews believed to be key determinants of sustainment approaches: (i) whether external support to sustain implementation is provided and (ii) whether such support strategies should (or could) be static or dynamic (Fig. 1). These two factors, we believe, are also primary considerations of agencies responsible for supporting the sustainment of health interventions. The proposed typology is intended to be a starting point for future work. A draft was initially put forward by author LW, circulated to co-authors (AS, AB, AH and NN), and iteratively refined through discussion. Early drafts were also presented for feedback to colleagues including those from public health policy agencies. The proposed typology definitions were also used to classify studies included in the review by Herlitz and colleagues [6]. All included studies where sustained EBI implementation was achieved could be classified in one of the typology categories, providing some early evidence of the utility and completeness of the working typology. Specifically, of the 24 studies that sustained their implementation of an EBI, 14 (58%) were classified as ‘*Self-Sustainment*’, nine (38%) as ‘*Static Sustainment Support*’, and one (4%) as ‘*Dynamic Sustainment Support*’. Additionally, we used the typology to classify 31 clinical and community-based health interventions undertaken by a health service in Australia for which one or more of the co-authors were engaged. The process identified a remarkable consistent distribution with 18 (58%) EBIs employing a ‘*Self-Sustainment*’ approach, 11 (35%) a ‘*Static Sustainment Support*’, and two (6%) ‘*Dynamic Sustainment Support*’ approaches. The clinical and community-based interventions included in the typology classification were selected as (i) they had previously been or are currently being delivered by an Australian Health Promotion Unit tasked with supporting the implementation and sustainment of government prioritised health promotion interventions in clinical (e.g. hospitals) and community (e.g. schools) organisations, and (ii) one or more of the manuscript authors have been engaged in the majority of included interventions and therefore familiar with the delivery. While providing a useful classification, approaches to sustainment are unlikely to be fixed. For example, interventions that are provided with external sustainability support (‘*Static Sustainment Support*’) may ‘*Self-Sustain*’ over time as organisational contexts become more favourable, capacity is built, and delivery becomes more routine.

### Self-Sustainment

‘*Self-Sustainment*’ approaches are those where no support is provided to organisations by external agencies, following a successful period of initial implementation. A ‘*Self-Sustainment*’ approach assumes the continued delivery of an EBI, at a level sufficient to accrue benefits will occur without external agency input. This may be most appropriate in instances where the evidence base supporting the beneficial effects of an EBI is well established and less likely that future research will discover the intervention is ineffective or harmful or that more effective or alternative interventions are identified in the short to medium term. This approach could be undertaken by agencies when funding or resources are limited, when priorities shift or when initial implementation efforts are judged to have sufficiently equipped organisations to sustain implementation. Although there is limited empirical evidence available, based on existing theoretical models and frameworks of sustainability [4, 5, 20], we hypothesise ‘*Self-Sustainment*’ approaches may be more likely to be successful when the target EBI is simple, inexpensive and well aligned with the implementing organisation’s needs, capacity, resources, values and broader context.

Given the increasing demands on health systems, and the need to maximise the benefits of scare health resources [22], EBIs that can be sustained using this approach are likely to have considerable merit from a health system perspective as their sustainment (and so benefits) are not dependent on ongoing external investment. The potential capacity for an EBI to be sustained by organisations should be considered early by policy makers and practitioners in the planning phases of selecting an EBI for delivery. Specifically, it may be prudent for external agencies without (or limited) resources or infrastructure to support ongoing sustainment, to identify early EBIs that are amenable to ‘*Self-Sustainment*’ [5]. To assist agencies to make this determination, future research could focus on identifying characteristics of interventions, contexts and initial implementation support strategies that influence ‘*Self-Sustainment*’ and the development of predictive models and ‘*Self-Sustainment*’ assessment tools. An example of a ‘*Self-Sustainment*’ approach is ‘*Crunch&Sip*’*.*

‘*Crunch&Sip*’ is a school-based program delivered in all primary schools in New South Wales (NSW), Australia, where teachers provide a daily opportunity in class for children to eat a piece of fruit and/or vegetables that they have brought from home and have a drink of water [23]. The program was first introduced in 2006, was well accepted by the school sector and achieved a high level of implementation across a large population of schools in Australia (approx. 80%) following an initial 11–15-month implementation period [23]. During initial implementation, schools received training, program resources, incentives, follow-up support, and implementation feedback from external health promotion unit. Given the relative simplicity of the intervention, its broad uptake across schools and evidence of its acceptability, local health promotion services considered planned ongoing support to schools to sustain implementation likely to be unnecessary. Data from a population wide monitoring survey of school environment found the program ‘*Self-Sustained*’, continuing to be implemented in > 90% of schools that had initially implemented it [23].

### Static Sustainment Support

‘*Static Sustainment Support*’ refers to the same pre-defined support strategy being provided to individual organisations by external agencies to support their sustained implementation of an EBI at a level sufficient to accrue benefit. The support could be time limited, such as the provision of a fixed number of monthly booster contacts or staff professional development, or ongoing, such as the use of monitoring and accreditation schemes. We acknowledge that there may be changes to the nature of a strategy provided to support sustainment, for example, changes in the amount of financial assistance provided to supporting sustainment of a program. We would suggest that such changes can be accommodated as part of a static sustainment approach, if the strategy continues to sufficiently address the same barrier (or facilitator) to sustainment. Deliberate changes to a strategy designed to address different barriers or alter (improve) its effects would be considered a dynamic approach. Some of the strategies employed in efforts to sustain EBI implementation may be similar to those used to facilitate its initial implementation. Ideally, however, there should be fewer strategies to support sustainment, and they should be less intensive than those required to successfully achieve initial implementation.

‘*Static Sustainment Support*’ may be particularly important when the support required to sustain implementation of an EBI is known, and relatively stable (not likely to change); when the implementing organisation does not, at least initially, have the capability to implement the EBI without such external support; and where external agencies have the resources and capacity to provide it. The continued provision of specialist equipment or devices, materials or technical assistance required for the ongoing implementation of an intervention is an example of ‘*Static Sustainment Support*’. The provision of external training to staff of an organisation to implement an EBI until the organisations’ internal training capabilities are re-oriented or established to meet this need would be another. ‘*Static Sustainment Support*’ may be more likely when the EBI that is to be sustained is more complex and where organisations face a greater number of barriers and are particularly vulnerable to decay if external support is withdrawn. It may also be more likely when the EBI itself is well supported by evidence and unlikely to change in the short to medium term.

Policy makers and practitioners may be compelled to provide external ‘*Static Sustainment Support*’ to sustain the implementation of EBIs in instances where there are no superior alternatives to replace the EBI or where the benefits of continued implementation are highly valued such as during short term public health crises. The research opportunities that may be most helpful in developing the evidence base for this approach include identifying the key components of an EBI that must be sustained in order for it to have an ongoing impact, determining what the determinants of the sustainment of these components are and assessing the effectiveness of strategies to improve the likelihood of sustained EBI implementation. An example of ‘*Static Sustainment Support*’ is ‘*Good Sports*’.

‘*Good Sports*’ is an Australian wide preventive health program that supports non-elite community-based sporting clubs from across a range of sporting codes to implement comprehensive alcohol management practices [24]. As part of the program, clubs progress through three levels of alcohol management accreditation [24]. This program was based on considerable formative evaluation and randomised trials. Sporting clubs represent challenging organisations to implement health interventions, given the volunteer and transit nature of their workforce and limited resources and infrastructure. Given such challenges, sporting clubs receive support by centralised support staff from the Alcohol and Drug Foundation to support initial and then ongoing implementation within an accreditation framework. A study compared existing centralised person-based support and a web-based support program that assisted clubs to undertake routine self-assessments and create an action plan and other prompts, online tools and resources. The study found at 25–27-month follow-up, sustainment of alcohol management practices was high in both groups (i.e. those that implemented the newer web-based support program and those with the existing centralised person-based support system) [24]. There was also no significant difference between intervention or control clubs for both the proportion of clubs implementing ≥ 10 of the 13 required practices (odds ratio: 0.53, 95% confidence interval [CI]: 0.04, 7.2; *p* = 0.63) or for the mean number of practices being implemented (mean difference: 0.10, 95% CI − 0.23, 0.42; *p* = 0.55) over the follow-up period [24].

### Dynamic Sustainment Support

‘*Dynamic Sustainment Support*’ describes a deliberate approach whereby external agencies provide resources or strategies to support EBI sustainment that continue to change over time which may occur, for example, as part of continuous quality improvement cycles (purposeful changes to strategies to support EBI implementation and sustainment occur to achieve continued improvement). ‘*Dynamic Sustainment Support*’ approaches may also be required if an EBI is complex and/or is evolving—necessitating ongoing changes to the support provided to sustain it. EBIs, for example, may change to remain aligned with evidence and changing standards of evidence-based practice or to improve their effectiveness, fit, efficiency, relevance, reach or acceptability [5]. This approach is consistent with ‘*Dynamic Sustainability Framework*’ perspectives [5].

Adaptation is a common and often necessary process of sustainment to improve the fit of an EBI or implementation and sustainment support strategies and would occur in each approach [4, 5]. From a ‘*Dynamic Sustainment Support*’ perspective, however, opportunities to adapt and modify are intentionally sought for the purpose of improvement, and processes are enacted to ensure that these occur repeatedly to guide incremental changes in strategies (and/or EBI) to support sustainment. Furthermore, while improvement could be an explicit goal of a ‘*Dynamic Sustainment Support*’ approach, improvement may also occur as part of all approaches. That is, improvement may naturally occur over time, with or without external sustainment support, as organisations and individuals involved in delivery of an EBI become more proficient.

‘*Dynamic Sustainment Support*’ may be best suited to circumstances where evidence regarding the effectiveness of interventions or their implementation and sustainment strategies is rapidly emerging and likely to change in the short term as research accumulates. Such circumstances create an ongoing tension for change to continually (re)align practice with evidence. ‘*Dynamic Sustainment Support*’ approaches are also likely to utilise different types of support strategies compared with static support approaches. For example, external support may focus on building an organisational climate of learning (i.e. implementing processes or approaches to foster organisational development by the continual reassessment of strategies) or evidence or implementation surveillance systems to identify when interventions and support strategies should be modified. It also requires ongoing resources and infrastructure to facilitate improvement processes, such as those described in the implementation, optimisation or improvement science literature (e.g. continuous quality improvement) [25]. External policy agencies may be particularly interested in ‘*Dynamic Sustainment Support*’ approaches for emerging health threats or priorities such as the prevention of e-cigarette use among non-smoking youth or COVID-19. Research opportunities to advance this approach may focus on the development of criteria to identify when and how best to modify implementation and sustainment strategies (or EBIs) to maximise their impact over time and the role of de-implementation in this processes and which strategies can be modified without sacrificing EBI effectiveness. An example of ‘*Dynamic Sustainment Support*’ is ‘*Physically Active Children in Education (PACE)*’.

‘*Physically Active Children in Education (PACE)*’ is a model of support delivered in primary schools (government, Catholic and independent) in six local health districts in NSW, iteratively developed and evaluated over 7 years to improve schools’ implementation of a mandatory physical activity policy [26–28]. The pilot study found that PACE was effective at increasing teachers’ implementation of the physical activity policy and led to a significant increase in students’ physical activity levels [26]. To enable the external agency responsible for supporting a large population of schools’ implement the policy, an optimisation framework was applied to PACE [25]. Sequential trials were undertaken testing and comparing different implementation strategies, and data on effectiveness, cost and acceptability from each phase were used to inform successive phases [28]. This process identified opportunities to incrementally improve PACE and resulted in a model of care, which, from the perspective of the external agency, was the most effective, cost-effective, efficient for delivery at-scale.

## Discussion

It is hoped this initial working typology may stimulate future research using more formal methods to further develop it. Nonetheless, we believe it represents a useful contribution towards the collective understanding of what approaches can be used to improve EBI sustainment from an external agency perspective such as policy and practice agencies responsible for improving healthcare. In all approaches, the typology assumes that the intervention is evidence-based and so when sufficiently implemented will yield health benefits and that these benefits will continue to accrue over the period in which implementation is sustained (see Additional file 2).

‘*Self-Sustainment*’ and ‘*Static Sustainment Support*’ approaches also assume they follow a period of successful improvement in implementation of the EBI at a level that warrants sustaining. That is, unless there is initial improvement in the implementation of an intervention that is sufficient to achieve health benefits, then sustainment is not warranted. We acknowledge that in many cases, implementation efforts may take a prolonged period of time in order to achieve levels of implementation that yield meaningful health improvements. We also appreciate such benefits are difficult to reliably anticipate and measure [29] as the effectiveness of interventions often attenuate (relative to those tested in efficacy studies) when delivered in the ‘real world’ [30]. Finally, regardless of the approach, EBI sustainment may not be achieved. Indeed, there are many examples where the implementation of the EBI has not been ‘*Self-Sustained*’, where implementation decay has occurred despite it being supported or where efforts to achieve ongoing improvement have been unsuccessful [6].

While providing a useful classification, approaches to sustainment are unlikely to be fixed. For example, interventions that are provided with external sustainability support (‘*Static Sustainment Support*’) may ‘*Self-Sustain*’ over time as organisational contexts become more favourable, capacity is built and delivery becomes more routine. In such circumstances, sustainment support may become redundant. Similarly, sustainment through continual improvement (‘*Dynamic Sustainment Support*’) may transition to be ‘self-sustained’, if, after a continual period of improvement, the evidence-base matures, and optimisation is achieved [25]. In this way, we acknowledge changes in approach are appropriate and in many instances inevitable, and so, the typology reflects an approach to sustainment of agencies charged with supporting it at a point in time. We have not proposed in the definition of ‘*Dynamic Sustainment Support*’ a specific frequency or time horizon for changes to an EBI. However, we suggest such approaches to sustainment are most appropriate in contexts where changes in core componentry of an EBI are anticipated in the short (or medium) term. Research to understand how approaches to sustainability evolve and transition is likely a fruitful area of future inquiry that may have significant policy and practice implications.

The proposed typology has a number of practice and research implications. Specifically, it may enable those responsible for supporting sustainment of EBIs to better consider which approach may be most suitable for their circumstance and develop strategies to align with this approach [31]. We anticipate, for example, that more dynamic support strategies such as facilitation (i.e. interactive problem solving and support) [31] may be particularly important for ‘*Dynamic Sustainment Support*’ [5, 32], while strategies targeting habit formation of organisations enabling them to better support the sustained static implementation of an EBI may be important components of ‘*Static Sustainment Support*’ approaches [32]. When external sustainment support is unavailable, the careful selection of EBIs for implementation that are amenable to ‘*Self-Sustainment*’ would appear prudent. Interestingly, most studies and services were assessed using the typology were classified as ‘*Self-Sustainment*’, while less than 10% were classified as ‘*Dynamic Sustainment Support*’ approaches. Such findings suggest that supporting the sustainment of an evolving EBI is an uncommon approach and may be due in part to perspectives of sustainment being relatively new or the logistic, feasibility or other challenges faced in supporting the implementation and sustainment of dynamic EBIs. It is also consistent with systematic reviews of the application of learning health systems for improvement in healthcare which identified very few examples of dynamic approaches to improvement occurring in practice despite their potential to optimise healthcare [33, 34].

The working typology may also help focus research for the field. A current criticism of sustainment studies is theories, models and frameworks employed to guide them do not included sustainment as the primary outcome [32]. The proposed working typology highlights the conceptual differences of sustainment as an outcome across approaches. For example, where an EBI is static, sustainment may be defined as the continued delivery of its fixed (core) components, whereas for dynamic EBIs, successful sustainment may be conceived as continued concordance with recommendations of ‘best practice’ guidelines as they evolve over time. Research will also be required to better understand the contexts best suited to the different sustainment approaches and assess the logistics, feasibility and other challenges that may be encountered in each approach supporting the sustainment of EBIs. Previous reviews have identified a range of barriers to EBI sustainment including the availability of equipment, resources and facilities, continued organisational executive support and staff turnover [6, 7]. It is unclear if and how important such barriers may be across the typology of approaches to sustainment. However, we anticipate variation in the relative importance of such factors between approaches. For example, ‘*Consolidated Framework for Implementation Research*’ in health constructs like a ‘learning climate’ may be more important for ‘*Dynamic Sustainment Support*’ compared with ‘*Self-Sustainment*’ approaches [35]. Further research is required to assess this hypothesis.

## Limitations

There are a number of limitations of this preliminary typology of sustainment approaches to be acknowledged. Firstly, similar to other published typologies, this initial typology was developed following discussion and reflection of the author team with academic colleagues and health policy makers and practitioners. While it provides an important conceptual basis, research methods such as consensus processes (e.g. Delphi) are recommended as part of future work to refine and strengthen the robustness of the typology. Secondly, as this is a preliminary typology of approaches to supporting the sustainment of EBIs, we have approached this from the perspective of an ‘agency’ tasked with supporting organisations to sustain programs—in our case a population health service. We have therefore included case studies for each typology approach that are within the Australian context we are familiar with and have delivered through the health service. However, as the typology evolves, there remains a critical need for further developmental work to investigate robust examples of each typology approach from a diverse range of contexts and settings to improve the generalisability of the typology. Lastly, although we used studies in one review by Herlitz et al. [6] to classify and examine studies according to each typology approach, as this typology evolves, it would be beneficial for future studies to apply the typology to other systematic reviews of EBIs. For example, coding studies conducted and sustained across a broader range of settings, with multiple independent coders, would more robustly test the applicability and suitability of the typology across contexts.

## Conclusions

Ensuring EBIs that are implemented and improving health continue to do so is critical if we are to maximise the returns on investment in clinical and public health services. The application of scientific methods to better understand sustainability is important to support policy makers and practitioners sustain the implementation of EBIs. This working typology contributes to this end by proposing definitions for three broad approaches to achieving sustained implementation, describing the contexts and circumstances where each may be most appropriate and outlining research and practice implications. While a more formal typology development and validation process employing more comprehensive cross disciplinary literature review of empirical and theoretical literature is warranted, it is envisaged the current working typology may provide a basis for this work and stimulate fruitful discussion to advance the field.

### Supplementary Information

**Supplementary Materials 1.**

**Supplementary Materials 2.**

## Data Availability

Not applicable.

## Abbreviation

EBI: Evidence-based intervention
