# Correction: An initial typology of approaches used by policy and practice agencies to achieve sustained implementation of interventions to improve health

**DOI:** 10.1186/s43058-024-00577-w

**Published:** 2024-04-04

**Authors:** Luke Wolfenden, Adam Shoesmith, Alix Hall, Adrian Bauman, Nicole Nathan

**Affiliations:** 1 School of Medicine and Public Health, College of Health, Medicine and Wellbeing, University of Newcastle, University of Drive, Callaghan, NSW 2308 Australia; 2 National Centre of Implementation Science (NCOIS), The University of Newcastle, Wallsend, NSW Australia; 3 Hunter New England Population Health, Hunter New England Local Health District, Wallsend, NSW Australia; 4 Hunter Medical Research Institute (HMRI), New Lambton Heights, NSW Australia; 5 School of Public Health, University of Sydney, Sydney, NSW Australia

**Correction** **:** **Implement Sci Commun 5: 21 (2024)**

**https://doi.org/10.1186/s43058-024-00555-2**

Following the publication of the original article [1], the authors reported an error with regard to Figure 1:


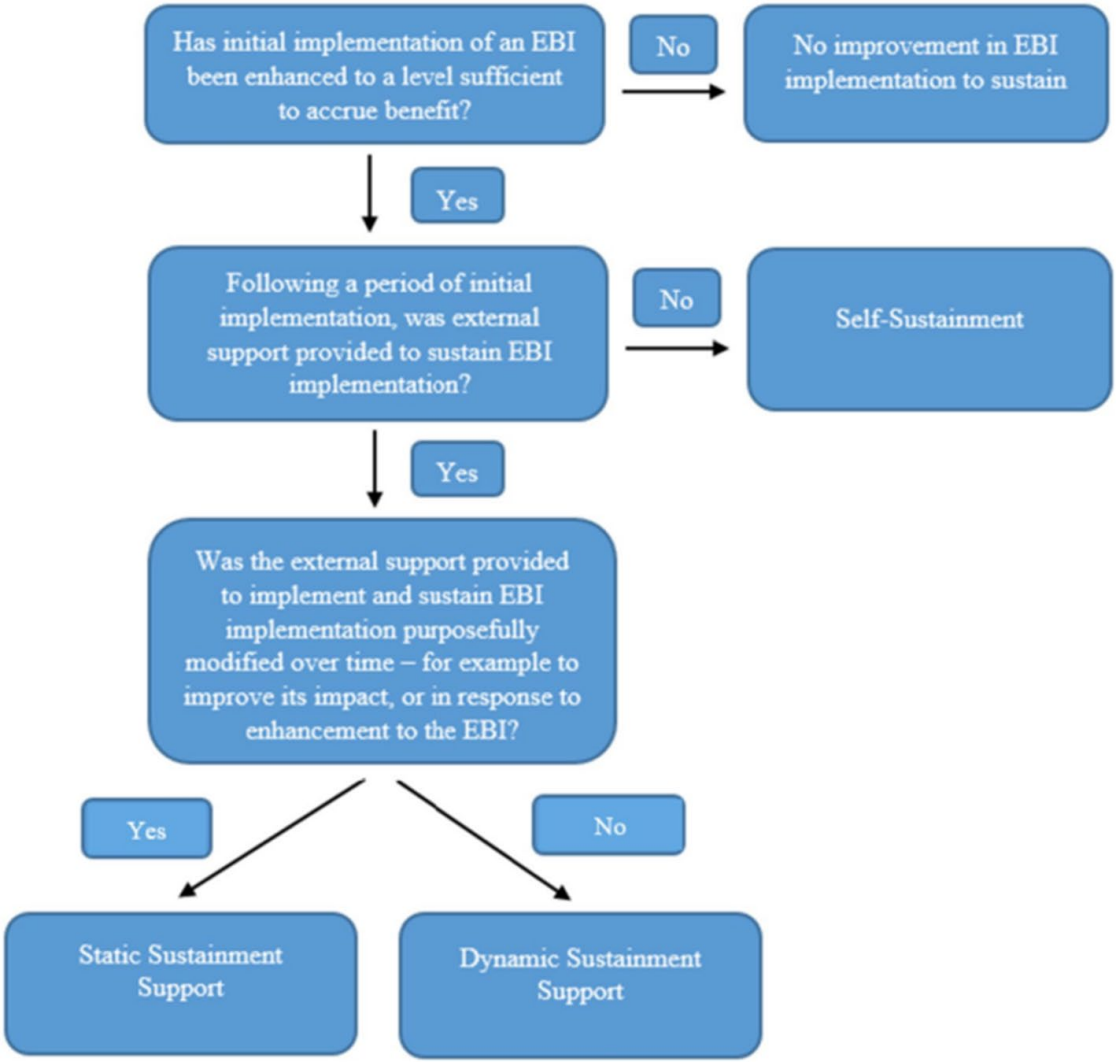


The “Yes” and “No” for the last decision needs to be switched, as shown in the correct Figure 1 below:


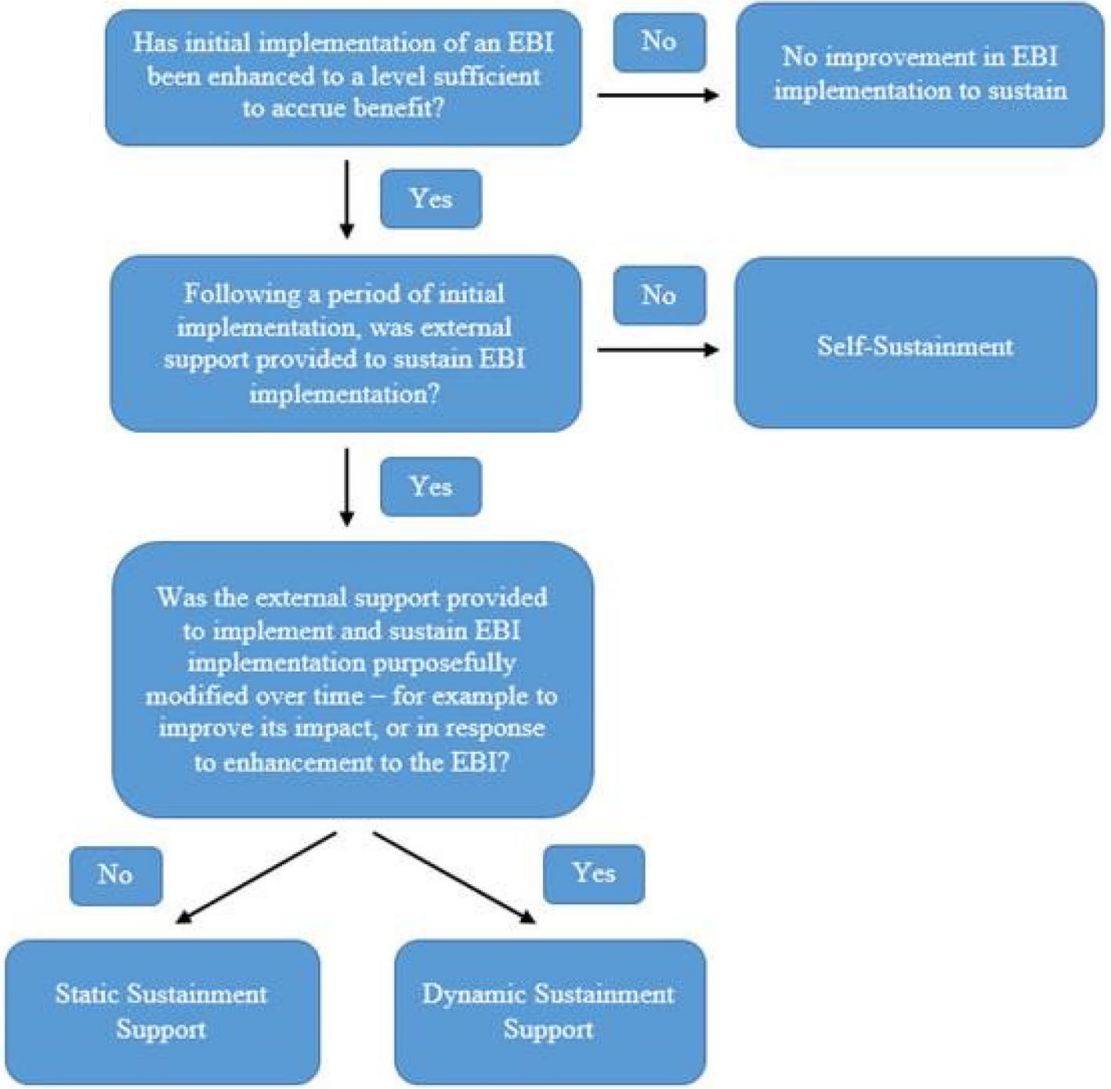


The original article has been corrected.
