# Supplementary material for: An initial typology of approaches used by policy and practice agencies to achieve sustained implementation of interventions to improve health
Source: Implement Sci Commun. 2024 Mar 5;5:21. doi: 10.1186/s43058-024-00555-2 (PMC10913259; doi:10.1186/s43058-024-00555-2)
Supplement: Supplementary file 1 — Supplementary Materials 1. [file 43058_2024_555_MOESM1_ESM.docx]

**Additional file 1.** Working typology of approaches to achieve EBI sustainment

| **Typology & Definition** | **Key Features** | | |
| --- | --- | --- | --- |
|  | **EBI Characteristics** | **External sustainment support** | **Contexts suggested to be most amenable for the sustainment approach** |
| ***Self-Sustainment***  The continued implementation of an EBI at a level sufficient to accrue benefit in the absence of external support. | • Fixed or dynamic EBI components. | • No substantive external support to facilitate sustainment of an EBI. | • When the characteristics of the EBIs are highly amenable to sustainment (e.g., simple, relative advantage, inexpensive).  • Where the required opportunity, capability and motivation for sustainment of the EBI exists within the implementing organisation.  • Where initial implementation is high and well integrated into the structures and processes of the implementing organisation.  • When there are no/or limited external resource available to support sustainment. |
| ***Static Sustainment Support***  The provision of defined (static) external support to an organization to facilitate the continued implementation of an EBI at a level sufficient to accrue benefit. | • Fixed core EBI components. | • External support to facilitate sustainment of an EBI that is static, and may be for a defined period or ongoing. | • Where the required opportunity, capability and motivation for sustainment does not exist within the implementing organisation.  • When the support to successfully sustain implementation of an EBI is known  • When EBIs and their sustainment support strategies have stable core components such as those supported by a mature evidence base, with established beneficial outcomes, and little chance of superior alternate interventions emerging in the short to medium term.  • When there are external resources available to support sustainment and/or where such resources may be available for at least a defined period of time. |
| ***Dynamic Sustainment Support***  The ongoing provision of external support to an organization that changes over time to best facilitate the continued implementation of an EBI at a level sufficient to accrue benefit. | • Dynamic core EBI components. | • External support to facilitate sustainment of an EBI that is dynamic (changes over time) and ongoing. | • Where the required opportunity, capability and motivation for dynamic approaches to sustainment does not exist within the implementing organisation.  • When core components of EBIs and their sustainment support strategies are likely to change, due to an emerging evidence-base, a high likelihood of superior interventions/core components being identified in the short to medium term or other factors.  • When there are ongoing external resources available to support sustainment including processes enabling to ongoing improvement and modifications to the provision of support strategies. |
