# Supplementary figures and images for: An initial typology of approaches used by policy and practice agencies to achieve sustained implementation of interventions to improve health
Source: Implement Sci Commun. 2024 Mar 5;5:21. doi: 10.1186/s43058-024-00555-2 (PMC10913259; doi:10.1186/s43058-024-00555-2)

**Additional file 2.** Health impact begins when programs are sustained


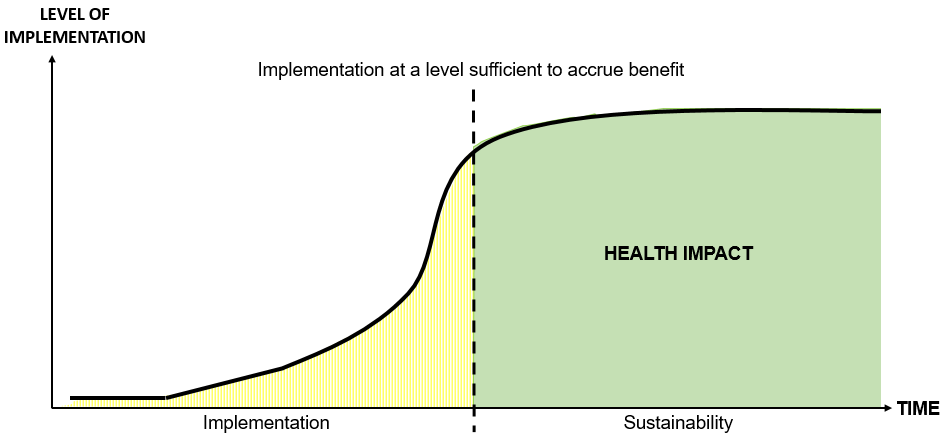

Supplement: Supplementary file 2 — Supplementary Materials 2. [file 43058_2024_555_MOESM2_ESM.docx]
